# The results of surgical treatment for pronation deformities of the forearm in cerebral palsy after a mean follow-up of 17.5 years

**DOI:** 10.1186/s13018-015-0251-3

**Published:** 2015-07-08

**Authors:** Goran Čobeljić, Stanislav Rajković, Zoran Bajin, Aleksandar Lešić, Marko Bumbaširević, Marko Aleksić, Henry Dushan Atkinson

**Affiliations:** Medical faculty University of Belgrade, Belgrade, Serbia; IOHB “Banjica” Belgrade, Serbia (Institute for orthopedic surgery “Banjica”), Belgrade, Serbia; North Middlesex University Hospital, London, N18 1QX UK

## Abstract

**Aim:**

This study evaluates the effects of three surgical procedures in the treatment of pronation deformities of the forearm in cerebral palsy patients; namely the transposition of pronator teres to extensor carpi radialis brevis muscle; and rerouting of the pronator teres muscle with or without pronator quadratus muscle myotomy.

**Methods:**

Sixty-one patients, 48 male/13 female, with a mean age of 17 years (5–41 years) were treated between 1971 and 2011. Pronator teres transposition was performed in 10, pronator rerouting in 35, and pronator rereouting with pronator quadratus myotomy in 16 patients. Ranges of motion, and assessments using the Quick Dash, Mayo Scoring, and Functional Classification system of upper extremity, were made before and after surgery. Mean follow-up was 17.5 years (3–41 years).

**Results:**

All three procedures led to significantly improved ranges of motion and upper limb function, with good/excellent results in 80 % of patients. Mean active supination improved from 10 ° (0–60 °) to 85 ° (30–90 °) (p < 0.001). There were significant improvements in Functional Classification system for the upper extremity scores (p < 0.003), Mean Quick Dash Scores improved from 58.41 (38.63–79.54) to 44.59 (27.27–68.18), and mean MEPS improved from 68 (30–85) to 84 (60–100) following surgery. All three techniques had statistically improved MEPS following surgery (p < 0.001); only the pronator teres muscle rerouting with pronator quadratus myotomy showed an improved Functional Classification system for the upper extremity score (p < 0.05); and only the pronator teres rerouting procedure showed an improved Quick Dash score (*p* < 0.05). There were no statistically significant differences in outcomes between different ages groups, and no significant differences between isolated pronator teres muscle rerouting were compared with those undergoing simultaneous treatment of carpal flexion and thumb adduction deformities (*p* > 0.05).

**Conclusion:**

Surgery is very effective in the management of pronation deformities of the forearm in patients with cerebral palsy. Isolated pronator teres rerouting is probably the most effective and simple technique. Adjunctive pronator quadratus myotomy does not lead to an improvement in the results and requires an additional surgical approach. There should be no age restriction to surgery, as all age groups appear to benefit from similar improvements in range of motion and upper limb function.

## Introduction

Pronation deformities of the forearm frequently occur in children suffering from cerebral palsy. They are caused by an imbalance between the stronger pronator teres and quadratus muscles, and weaker supinator muscles. They are often overlooked by both parents and physicians whose attention is usually drawn more to the deformities affecting the lower extremities and the associated problems with gait and the ability to sit.

However, the significance of these pronation deformities is not small. Aside from the aesthetics, a pronated forearm position interferes with normal hand and finger use, particularly as patients are not able to see their own palms. This precludes many important social and functional activities including handshaking, face washing, clapping etc. A pronated forearm position also exacerbates flexion deformities in the hand, and when supination is significantly restricted, patients often have to compensate through other body and shoulder movements, frequently adopting bizarre postures [1, 2].

Patients also run the risk of radial head dislocations, most commonly in a posterolateral direction. A dislocated radial head can further limit forearm extension and supination, and during skeletal growth often leads to posterior angulation of the proximal ulna and tenting under the skin [3].

The treatment of these deformities is complex, and they are often managed through both non-operative and operative means. Non-operative treatments include physical therapy, the use of splints and casts, and botulinum injections into the pronator muscles. Corrective orthotics and casts usually enclose the whole of the upper limb and can further impede function [4]. The surgical management can be broadly divided into five groups though it is far from clear as to when or which procedures to undertake. Commonly performed operations include:

1. Procedures involving the pronator teres muscle. These can be releases at proximal or distal ends; elongation of the muscle; transposition of the pronator teres to the extensor carpi radialis brevis muscle; rerouting of pronator teres [1, 5, 6];
2. Myotomy of the pronator quadratus muscle [7];
3. Surgery to other muscles whose function is not primarily pronation. Flexor carpi ulnaris transposition to extensor carpi radialis brevis; brachioradialis rerouting [8, 9];
4. Various combinations of 1,2 and 3;
5. Radial (rotational) osteotomy [10].

The aim of this study was to evaluate the outcomes of three operative procedures: transposition of pronator teres muscle to extensor carpi radialis brevis and rerouting of pronator teres with or without pronator quadratus myotomy. A second aim was to determine whether the surgery to correct the pronation deformity led to better hand usage and better overall upper limb function.

## Material and methods

Between 1971 and 2011, ninety-two patients with cerebral palsy underwent surgery to correct their forearm pronation deformities at the Institute for Orthopedic Surgery “Banjica” in Belgrade. This is a retrospective review of 61 of these patients; the remaining 31 patients did not meet the inclusion criteria. Ethical approval was sought and given by Belgrade University Medical School Ethics Board for this retrospective review, and all 61 patients and/or legal guardians gave their informed consent to be included in the study.

Inclusion criteria included patients with a pronation deformity of the forearm who had previously only had non-operative treatment, patients with spastic hemiplegia, patients greater than 5 years of age and those with a minimum follow-up period of 3 years. Exclusion criteria included non-cerebral palsy patients and non-spastic cerebral palsy patients with a pronation deformity due to other reasons, patients with an IQ less than 70, and patients with disrupted hand sensibility (and inability to recognise either the form or quality of an object). Patients with incomplete hospital records or poorly recorded clinical data were also excluded.

There were a total of 48 (78.7 %) male and 13 (21.3 %) female patients, and the mean age at the time of surgery was 17 years (5–41 years). Thirty-nine involved the right upper limb (63.5 %) and 22 the left (36.5 %). The indications for surgery were patients who had a restricted active forearm supination of less than 60 °, from a position of full pronation, where passive supination of 90 ° was possible [4, 7, 11].

**Fig. 1:**
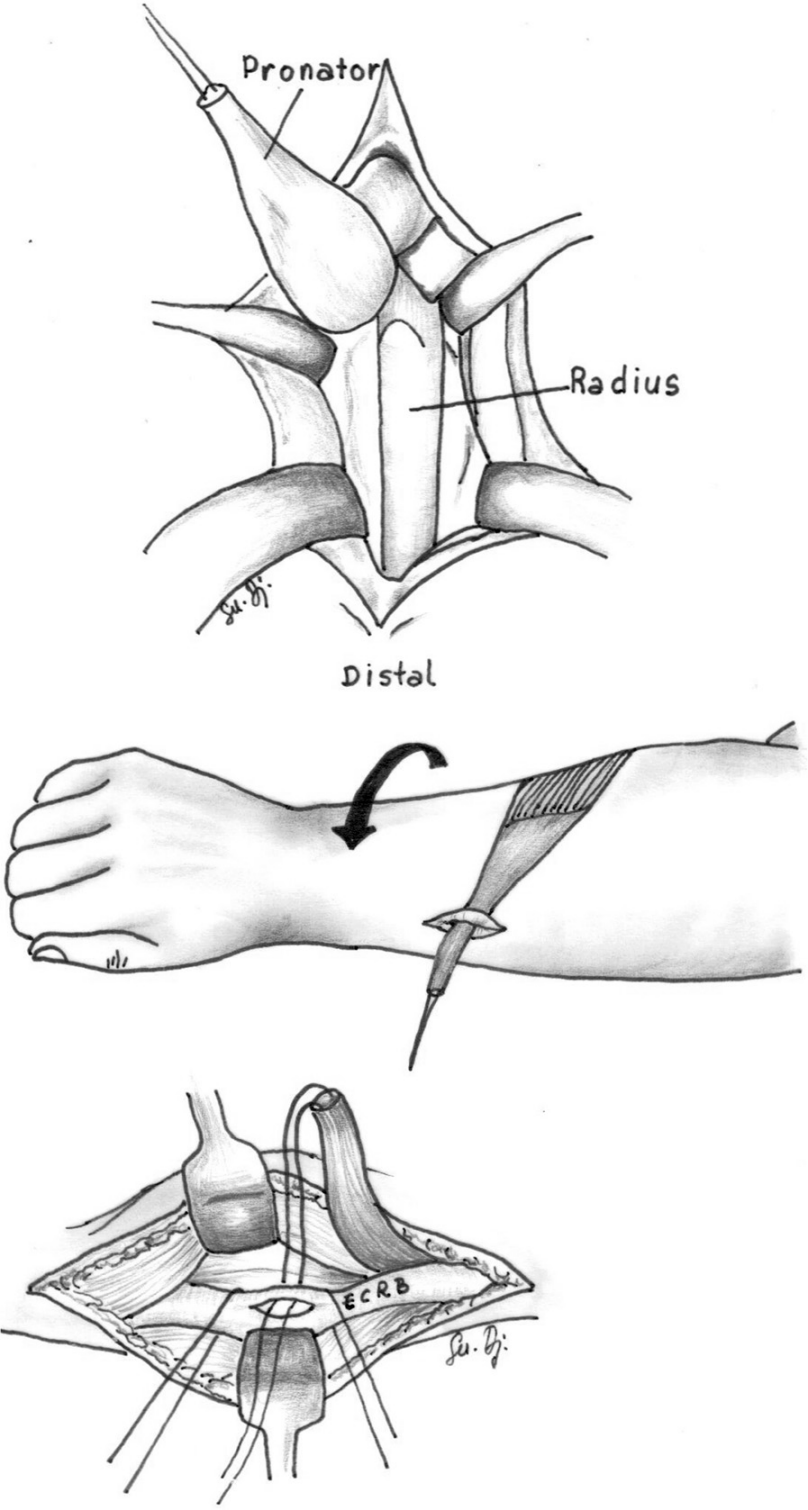
A schematic of a pronator teres to extensor carpi radialis brevis transposition

**Fig. 2:**
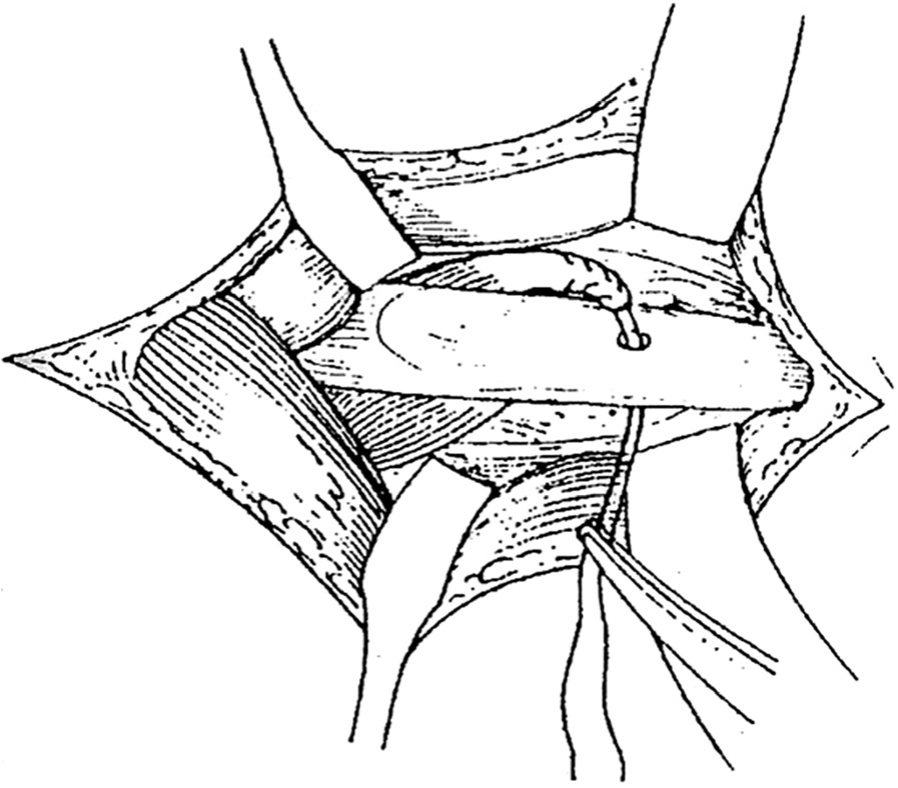
A schematic of a rerouting of pronator teres

**Fig. 3:**
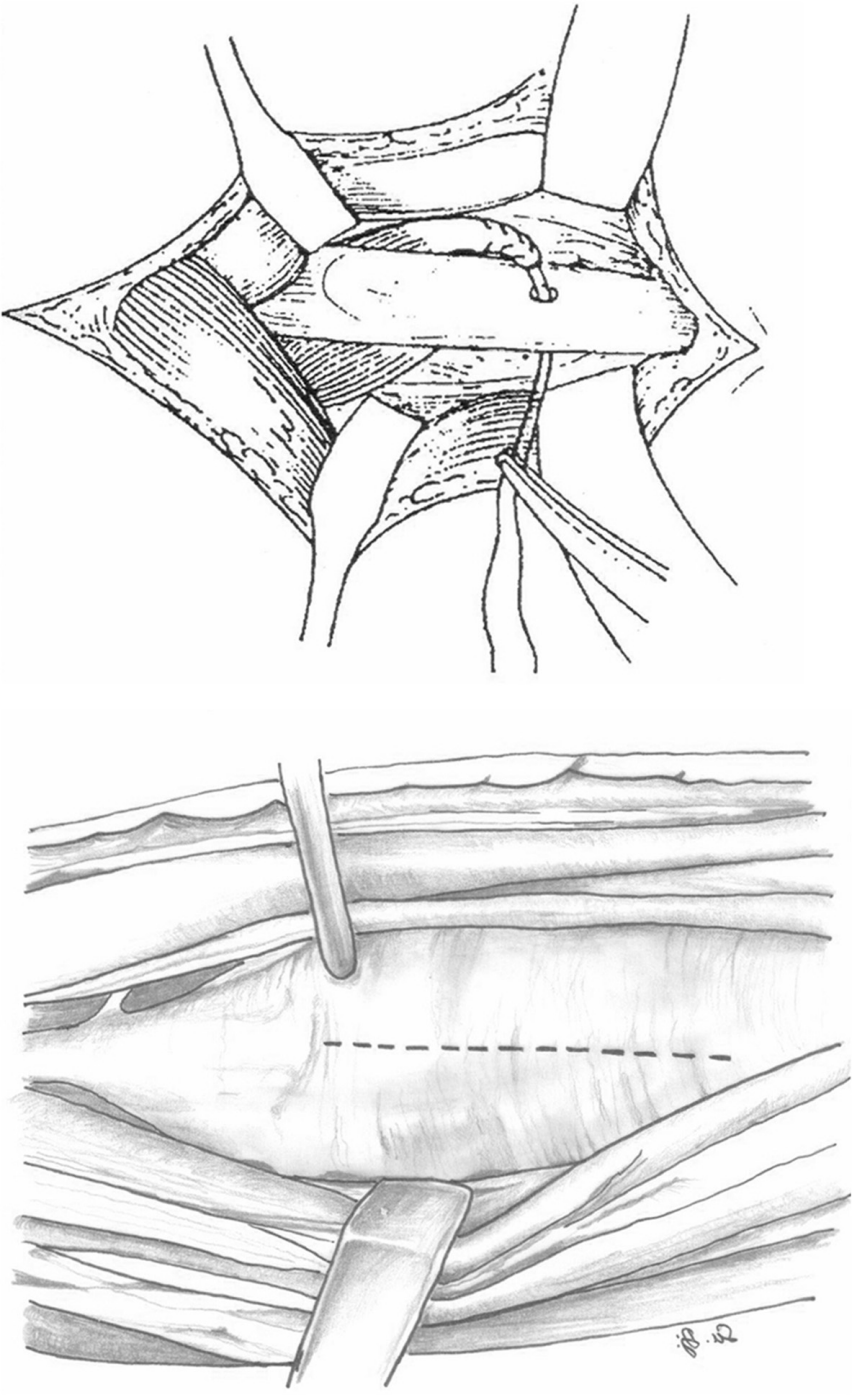
A schematic of a rerouting of pronator teres with a pronator quadratus myotomy

Patients either underwent a pronator teres to extensor carpi radialis brevis transposition (Fig. 1) [5], a rerouting of pronator teres (Fig. 2) [6], or a pronator quadratus myotomy combined with rerouting of the pronator teres (Fig. 3) [7]. The postoperative management was the same for all three procedures. The upper limb was immobilised in a long arm cast for 3 weeks with the elbow flexed at 90 ° and in a position of maximal forearm supination. After 3 weeks, the casts and stitches were removed and rehabilitation was commenced. Passive and active elbow movement was encouraged, and the forearm then continued to be gently manipulated/stretched in order to maintain the full supination.

Pronator teres transposition was performed in 10 cases, pronator teres rerouting in 35 cases, and combined pronator quadratus myotomy and pronator teres rerouting in 16 cases. Eighteen patients also underwent concurrent/simultaneous hand and finger flexor muscle elongation, 13 had flexor carpi ulnaris muscle to extensor digitorum communis muscle transpositions in order to correct flexion deformities of the hand, and 6 had correction of adduction deformities of the thumb. Surgery for hand and finger flexion deformities was indicated if active extension of the fingers was only possible with the hand flexed over 20 °. Adduction deformity of thumb was corrected through myotomy of the first dorsal interosseous muscles and tenotomy of the adductor pollicis muscle.

Active supination and pronation ranges of motion were recorded, and the surgical results were assessed according to the Functional Classification system for the upper extremity [4], the Quick Dash Score (to assess upper extremity function [12]) and the Mayo Elbow Performance Score (MEPS) (to asses elbow function [13]). The Functional Classification system for the upper extremity is a 0–5 6-point scoring system (0 being worst and 5 best function); the Quick Dash Score is a 0–100 system (a lower score shows a higher functional ability), MEPS is 5–100 system (scores under 60 correspond to poor, 60–74 to fair, 75–89 to good and above 90 to excellent results). The assessments were performed before and after operative treatment by three different investigators who did not perform the surgery and were not familiar with the results. The patients were assessed at six monthly intervals over the first three post-operative years and then at one yearly intervals. One should point out that historic patient clinical and disease data were mined from the case notes and other hospital records in order to calculate the initial functional and performance scores. Quick Dash and MEPS scores were prospectively measured from 2001, and Functional Classification score data was collected prospectively from 2008. Where these data were not available, then patients were excluded from the series.

The results of surgery were statistically analysed individually and with respect to one another using Wilcoxon t-signed rank test, Student *t*-test and Kruskal-Wallis tests. Statistical significance was set at *p* < 0.05.

## Results

After a mean follow-up of 17.5 years (3–41 years), the whole patient cohort was found to have had significant improvement in their ranges of motion from a mean preoperative active supination of 10 ° (0–60 °) to a mean postoperative supination of 85 ° (30–90 °) (*p* < 0.001). There was no statistically significant difference between the three operative techniques when these ranges of motion were analysed separately, i.e. no particular operative procedure was found to have any particular advantage in achieving an improved level of active supination and pronation movement. All patients had a preoperative full active pronation of 90 °, and postoperative average pronation was 83 ° (40–90 °).

When all three surgical groups were pooled, all three scoring systems registered improvements following surgery. The Functional Classification system for the upper extremity found a statistically significant improvement (*p* < 0.003), the Mean Quick Dash Scores improved from 58.41 (38.63–79.54) to 44.59 (27.27–68.18), and mean MEPS improved from 68 (30–85) to 84 (60–100) following surgery. A breakdown of these MEPS found that two cases had poor, 42 fair and 17 good function pre-surgery. There were no poor, 12 fair, 25 good and 24 excellent outcomes following surgery. Good or excellent results were achieved in 80 % of the surgically treated patients.

**Fig. 4:**
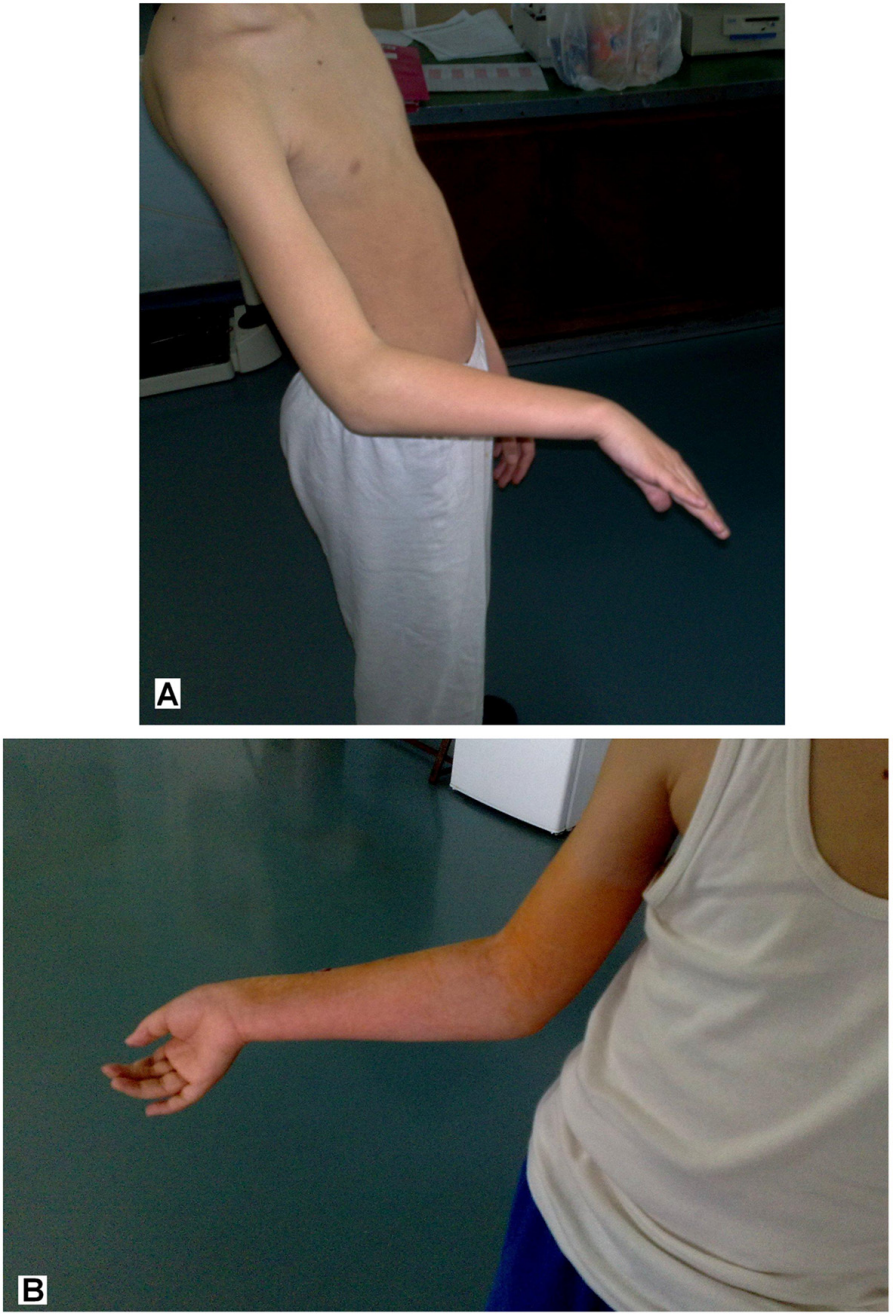
**a** Pronation deformity of the right forearm in a spastic form of cerebral palsy before surgery. **b** Following corrective surgery by rerouting the pronator teres muscle

When analysing each individual surgical procedure separately, it was found that all three surgical techniques had statistically improved MEPS following surgery (*p* < 0.001), and there were no tangible differences between the three procedures. However, only the pronator teres muscle rerouting with pronator quadratus myotomy showed a statistically improved postoperative result using the Functional Classification system for the upper extremity (*p* < 0.05), and similarly only the pronator teres rerouting procedure showed a statistically improved Quick Dash Score postoperatively (*p* < 0.05) (Fig. 4a, b).

The patients were further subdivided into three age ranges, 5–11 years (17 patients), 12–18 years (16 patients) and 19 years and over (28 patients), and it was found that there were no statistically significant differences in the surgical outcomes between these ages groups.

There were no statistically significant differences in any of the assessment systems when the results of isolated pronator teres muscle rerouting were compared with those 13 patients also undergoing simultaneous treatment of their carpal flexion and thumb adduction deformities (Mann–Whitney test >0.05). The other subgroups of combination procedures were too small to yield any meaningful analyses.

There were no surgical complications.

## Discussion

There is no clear consensus over how to optimally manage pronation deformities of the forearm in cerebral palsy patients. However, though there are some reports of positive outcomes of non-operative treatment by cast alone [14], most authors favour operative treatment [2, 7] and non-operative management should now probably only really be considered as an adjunct to a surgical correction.

Many surgical procedures have been described to treat these deformities, and there remains no agreement about when or which procedure to use. Many authors favour rerouting of the pronator teres muscle [6, 11, 15] and have concluded that this is better than pronator teres tenotomy alone [16]. Some have shown good results with a transposition of the pronator teres muscle to the hand extensor muscles, with or without a pronator quadratus myotomy [5, 7], while others have concluded that a transposition of the flexor carpi ulnaris to the extensor carpi radialis brevis muscle, with or without a lengthening of pronator teres, and rerouting of brachioradialis muscle give the best results [8, 9, 17, 18].

This study has shown that surgery is very effective in the management of pronation deformities of the forearm in patients with cerebral palsy. All the three procedures investigated (pronator teres muscle transposition, and rerouting of pronator teres with or without pronator quadratus myotomy) led to significantly improved ranges of motion and upper limb function. This was true for all the patient age groups, including those aged over 19 years. There were no differences in outcomes between isolated forearm procedures and those combined with other deformity corrections.

There was no significant difference in range of motion or MEPS between the three procedures; only pronator teres rerouting had statistically improved Quick Dash Scores, and only pronator teres muscle rerouting with pronator quadratus myotomy showed a statistically improved Functional Classification system for the upper extremity scores.

These mixed functional outcomes highlight the limitations in the scoring systems that were used and the likely need for clearer discriminators; certainly, the Quick Dash and Mayo Scores are not specific to patients with neurological disorders. One can speculate whether clearer distinctions might have been found between the procedures using other scoring systems such as MALUF (Melbourne Assessment of The Unilateral Upper Limb Function Test), MACS (Manual Ability Classification System), SHUEE (Shriner’s Hospital Upper Extremity Evaluation), or MHC (Modified House Functional Classification system) which is used for patients with cerebral palsy (though only in the 3–18 year age range) [19–22].

## Conclusion

Surgery is very effective in the management of pronation deformities of the forearm in patients with cerebral palsy. It is difficult to draw any stronger conclusions as to which of the surgical techniques in this study might be best, and indeed, their results are similar.

However, the authors would favour performing a pronator teres rerouting in isolation as this is a simple and yet very effective procedure, and an adjunctive pronator quadratus myotomy does not appear to lead to any improvement in the results. Moreover, the adjunctive quadratus myotomy also requires an additional surgical approach, with its associated morbidity, and we therefore do not recommend it. There should not be any age restriction to surgery, and each of the age sub-groups appeared to benefit from similar improvements in range of motion and upper limb function.
